# Robust *in vitro* assay for analyzing the neutralization activity of serum specimens against hepatitis B virus

**DOI:** 10.1080/22221751.2019.1619485

**Published:** 2019-05-25

**Authors:** Ya-Li Zhang, Ying Gao, Jia-Li Cao, Jing-Hua Zhao, Tian-Ying Zhang, Chuan-Lai Yang, Hua-Long Xiong, Ying-Bin Wang, Shan-Hai Ou, Tong Cheng, Chang-Rong Chen, Quan Yuan, Ning-Shao Xia

**Affiliations:** a State Key Laboratory of Molecular Vaccinology and Molecular Diagnostics, School of Public Health, Xiamen University, Xiamen, People’s Republic of China; b School of Life Science, National Institute of Diagnostics and Vaccine Development in Infectious Diseases, Xiamen University, Xiamen, People’s Republic of China; c Xiamen Blood Service, Xiamen, People’s Republic of China; d Hainan Health Disseminate Centre, Haikou, People’s Republic of China; e Natural Medicine Institute of Zhejiang Yangshengtang, Hangzhou, People’s Republic of China; f Xiamen Haicang Hospital, Xiamen, People’s Republic of China

**Keywords:** Hepatitis B virus, cell-based assay for neutralization activity against HBV, hepatitis B neutralizing antibodies, hepatitis B surface antibody, hepatitis B core antibody

## Abstract

Anti-HBs is a well-known marker of protective capability against HBV. However, little is known about the association between the qAnti-HBs determined by immunoassays and the neutralization activity (NAT) derived from functional assays. We developed an *in vitro* assay for direct measurement of the NAT of human sera. The new assay was highly sensitive, with an analytical sensitivity of 9.6 ± 1.3 mIU/mL for the HBIG standard. For serum detection, the maximum fold dilution required to produce ≥50% inhibition (MDF_50_) of HBV infection was used as the quantitative index. *In vitro* NAT evaluations were conducted for a cohort of 164 HBV-free healthy individuals. The results demonstrated that the NAT positively correlated with the qAnti-HBs (*R*^2^ = 0.473, *p* < 0.001). ROC analysis indicated that the optimal cutoff value of the qAnti-HBs to discriminate significant NAT (MDF_50_ ≥ 8) was 62.9 mIU/mL, with an AUROC of 0.920. Additionally, we found that the qAnti-HBc was another independent parameter positively associated with the NAT (*R*^2^ = 0.300, *p* < 0.001), which suggested that antibodies against other HBV proteins generated by previous HBV exposure possibly also contribute to the NAT. In summary, the new cell-based assay provides a robust tool to analyse the anti-HBV NAT.

**Abbreviations:** HBV: Hepatitis B virus; HBsAg: Hepatitis B surface antigen; Anti-HBs: Hepatitis B surface antibody; HBeAg: Hepatitis B e antigen; Anti-HBc: Hepatitis B core antibody; qAnti-HBs: quantitative hepatitis B surface antibody; qAnti-HBc: quantitative hepatitis B core antibody; qHBeAg: quantitative hepatitis B e antigen; NAT: neutralization activity; HBIG: hepatitis B immune globulin; NTCP: Na^+^-taurocholate cotransporting polypeptide; IRES: internal ribosome entry site; ccHBV: cell culture derived hepatitis B virus; GE/cell: genome equivalent per cell; MOI: multiplicity of infection; Dpi: day post infection; HepG2-TetOn: a HepG2-derived cell line that expresses the doxycycline-regulated transactivator; ROC: receiver operating characteristic curve; AUROC: area under receiver operating characteristic curve; LLOQ: the lower limits of quantification; MDF_50_: the maximum fold dilution required to produce ≥50% inhibition; IC50: half maximal inhibitory concentration

## Introduction

Hepatitis B virus (HBV) infection remains a global health challenge. According to a recently reported modelling study, it was estimated that the global prevalence of HBsAg in 2016 was 3.9%, corresponding to approximately 290 million individuals with chronic HBV infection [1]. HBV infection can induce acute, fulminant, or chronic hepatitis, liver cirrhosis (LC), and hepatocellular carcinoma (HCC). More than 700,000 people die due to diseases associated with HBV infection [2]. Immunoprophylaxis via active immunization with preventive hepatitis B (HB) vaccines and/or passive immunization with hepatitis B immunoglobulin (HBIG) are effective in preventing new HBV infection [3]. Although several drugs, including interferons and nucleos(t)ide analogs, have been approved for HBV treatment, a functional cure for established chronic HBV infection is still difficult to achieve [3]. Therefore, prevention of HBV infection by vaccine immunization is regarded as the best way to eliminate HBV-related diseases [4].

Recombinant hepatitis B small surface antigen (S-HBsAg, 226 aa) particles are used as the immunogen in most commonly used HB vaccines to induce preventive anti-HBs antibodies. Antibodies raised by vaccination recognize the conformation-dependent “a” antigenic loop (AGL) comprising aa110-aa160 of sHBsAg [5]. According to previous studies, the AGL region is responsible for the initial interaction between the virus and cell surface heparin sulfate proteoglycans [6,7]. Therefore, AGL-directed antibodies generally exhibit potent neutralization activity similar to that of antibodies against the viral cellular receptor (NTCP) binding site (RBD) in the PreS1 region [8,9]. During the past three decades, the safety and effectiveness of the sHBsAg-based vaccine was successfully demonstrated in protecting people from HBV infection and HBV-related diseases worldwide [10]. Antibodies specific for sHBsAg raised by vaccination were considered as the only neutralizing antibody among these humans who did not have exposure to HBV infection. In contrast, individuals who recovered from past HBV infection may have additional neutralizing antibodies recognizing PreS1 and/or PreS2. For HB vaccine immunization, an adequate anti-HBs antibody response is widely considered to be a titre greater than or equal to 10 mIU/mL [11], and a booster immunization is recommended when the titre falls below 10 mIU/mL. However, due to the absence of a robust *in vitro* HBV infection assay, the association between the anti-HBs binding titre and the neutralization activity (NAT) remains largely unknown. Moreover, it is unclear whether there is any NAT difference in between anti-HBs antibodies derived from vaccination and resolved natural infection. To address these issues, we developed a doxycycline (dox)-inducible NTCP-overexpressing cell line that supports high-efficiency *in vitro* HBV infection and therefore enables direct measurement of the neutralization activity (NAT) of human serum specimens. By using this new assay, we systematically investigated the associations between serological markers and NAT titres in a well-characterized cohort.

## Materials and methods

### Plasmids and cells

The cDNA of human sodium taurocholate cotransporting polypeptide (hNTCP) was ligated with an IRES-driven mCherry (IRES-mCherry) reporter by PCR. The hNTCP-IRES-mCherry DNA fragment was subsequently inserted into a pLenti-CMVTRE3G-eGFP (Addgene 27570) vector. Recombinant lentiviruses were produced to transduce HepG2-TetOn cells (Clontech Laboratories, Otsu, Japan). Stably transduced cells were obtained by flow cytometry cell sorting (FACS) on a BD FACSAria III and further cultured in the presence of puromycin (3 μg/mL). After 3 weeks of selection, puromycin-resistant cell clones were isolated for further evaluation of dox-inducible mCherry expression and PreS1 peptide binding. For the PreS1 peptide binding assay, cells were incubated with N-terminal myristoylated HBV PreS1 (amino acids 2–48) peptide with C-terminal FITC labelling (customized from Sangon Biotech, Shanghai, China). One hour after incubation, cells were washed 2–3 times with PBS and analyzed by flow cytometry.

### *In vitro* HBV infection assay

Cell culture-derived HBV (ccHBV) viral stocks for the infection assay were obtained from the culture medium of HepAD38 cells as previously described [12,13]. Infectious HBV particles were concentrated from culture supernatants by precipitation with 5% PEG and were then resuspended in DMEM supplemented with 10% fetal bovine serum (FBS). The viral titre (genome equivalent, GE) was determined by a quantitative PCR assay. For HBV infection, HepG2-TetOn-NTCP cells were pretreated with 3 μg/mL dox in culture medium for 3–4 days to induce NTCP expression. Subsequently, ccHBV was incubated with dox-treated cells at a defined multiplicity of infection (MOI) in the presence of 4% PEG 8000 for 24 h, and the cells were then washed three times with PBS and further cultivated with dox-containing fresh culture media. During the culture of HBV-infected cells, the culture media were collected and refreshed every 2 or 3 days thereafter.

### Measurement of NAT in HBIG and human serum samples

Hepatitis B immune globulin (HBIG) was used as a standard sample to evaluate the sensitivity and accuracy of the NAT assay. For the assay, diluted HBIG was preincubated with ccHBV in Dox- and PEG-containing culture medium for 1 h, and then the mixture was then added to dox-treated HepG2-TetOn-NTCP cells to perform the infection assay. For serum specimen tests, the samples were first centrifuged at 13,000 × g for 15 min and sterilized by filtering through a 0.22 μm filter before incubation with ccHBV and conduction of cell-based experiments. It should be noted that if the specimens were initially prepared and stored in sterilized tube, the filtration sterilization may be not required. To our experience (data not shown), serum filtering through a 0.22 µm filter did not influence its NAT titre determination.

### Immunoassays for HBV markers

For human serum sample quantitative anti-HBs (qAnti-HBs) measurement, two commercial immunoassays were used: one was a chemiluminescent microparticle immunoassay (Archetect i2000, Abbott Diagnostics, Abbott Park, IL, USA), and the other was an ELISA kit (Wantai Biological Pharmacy, Beijing, China). The qAnti-HBc level was measured using a newly developed double-sandwich immunoassay (Wantai Biological Pharmacy, Beijing, China) as previously described [14]. The levels of the two antibody markers were expressed in mIU/mL (qAnti-HBs) or IU/mL (qAnti-HBc) calibrated using the WHO standard [15]. Hepatitis B surface antigen (HBsAg) in the culture supernatants of HBV-infected cells was quantitatively determined by a microplate chemiluminescence HBsAg assay (Wantai Biological Pharmacy, Beijing, China) calibrated using the WHO HBsAg standard. For hepatitis B e antigen (HBeAg) measurements, an ELISA-based kit was used (Wantai Biological Pharmacy, Beijing, China), and values were expressed with reference to the China National Clinical Unit (Ncu/mL). The lower limits of quantification (LLOQ) of the assays in this study were 0.08 IU/mL for qAnti-HBc, 10 mIU/mL for qAnti-HBs, 0.05 IU/mL for HBsAg and 0.1 Ncu/mL for HBeAg.

### Subjects and cohorts

A cohort consisting of 164 volunteer blood donors was recruited from the Xiamen Blood Service from 15 to 19 October 2014. All subjects were negative for HBV (negative in both HBsAg and HBV DNA tests), HCV/HIV (negative in both anti-HCV/HIV antibody and HCV/HIV RNA tests), and anti-*Treponema pallidum* and had alanine aminotransferase (ALT) levels lower than the upper limit of normal (40 U/L). Serum samples from these donors were stored at −20°C until analyzed. The study was approved by the Institutional Review Board of the School of Public Health at Xiamen University in accordance with the Declaration of Helsinki. Informed consent was obtained from each subject.

### Statistical analysis

Continuous variables such as age, qAnti-HBs, and qAnti-HBc were compared using an independent samples t-test. Categorical variables such as sex and blood type were compared using the Mantel-Haenszel *χ*^2^ test or Fisher’s exact test. Linear regression models were used for correlation analyses for NAT and other parameters, such as qAnti-HBs and qAnti-HBc (Pearson’s correlation). Statistical analysis was performed using SPSS (Statistical Package for the Social Sciences) ver. 21.0 software (SPSS, Chicago, IL, USA). All statistical analyses were based on 2-tailed hypothesis tests with a significance level of *p* < 0.05.

## Results

### Generation and characterization of HepG2-TetOn-NTCP cells for HBV infection

**Figure 1.**
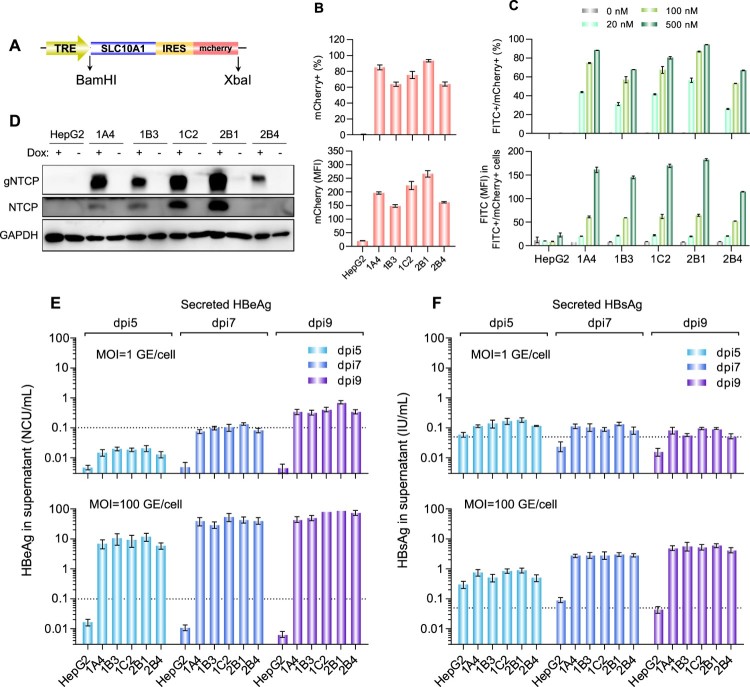
Establishment and characterization of NTCP-reconstituted HepG2 cells for *in vitro* HBV infection. (A) A schematic diagram depicting the dox-inducible NTCP expression cassette. The NTCP-IRES-mCherry cassette was cloned into the pLenti-CMVTRE3G-eGFP (Addgene 27570) vector between the BamHI and XbaI restriction sites. (B) Flow cytometric analyses of the dox-induced mCherry expression of the different HepG2-TetOn-NTCP cell lines. The upper panel showed the percentage of mCherry+ cell population, and the lower panel showed the mean fluorescence intensity (MFI) of mCherry+ cell population of various cell lines. (C) Flow cytometric analyses of the dox-induced PreS1-peptide (FITC conjugated) binding capability of the different HepG2-TetOn-NTCP cell lines. The upper panel presented the dose-dependent comparison on the FITC/mCherry double positive percentage of different cell lines, and the lower panel presented the FITC MFI of FITC+/mCherry+ cell populations of various cell lines when binding with myr-PreS1-FITC peptide at different dosage (500, 100 and 20 nM). (D) Western blots for NTCP expression in the different HepG2-TetOn-NTCP cell lines with or without dox induction. The NTCP was detected by a commercial rabbit polyclonal antibody against NTCP (Thermo Fisher Scientific, PA525614). (E, F) Detections of HBeAg (E) and HBsAg (F) in culture supernatants of different HepG2-TetOn-NTCP cell lines following infection with HBV at 1 GE/cell (upper panel) or 100 GE/cell (lower panel) at dpi 5, 7 and 9. The FACS analyses, PreS1-peptide binding assay and western blots were performed at 4-day after dox-treatment (3 μg/mL). The data in panels B, C, E and F are expressed as the means ± SDs. The limits of quantitation for the HBeAg and HBsAg assays used in our study were 0.1 Ncu/mL and 0.05 IU/mL, respectively. TRE is a tetracycline response promoter; mCherry is a bright red fluorescent protein; SLC10A1, solute carrier family 10 member 1, is the gene name of the NTCP protein; IRES is the internal ribosome entry site; gNTCP, glycosylated NTCP.

To generate HBV-susceptive cells, a dox-inducible NTCP expression cassette with an IRES-driven mCherry reporter (Figure 1(A)) was stably transfected into HepG2-TetOn cells vial lentiviral transduction. After puromycin selection and single-cell cloning, we obtained 5 stable HepG2-TetOn-NTCP cell lines (HepG2-1A4, HepG2-1B3, HepG2-1C2, HepG2-2B1 and HepG2-2B4). After dox-induction, all 5 cell lines expressed detectable mCherry and showed specific PreS1-peptide (FITC-conjugated) binding capability in a dose-dependent manner (Supplemental Figure 1). Flow cytometry analyses revealed that the PreS1-peptide binding signals of these cells positively correlated with their mCherry expression levels (Supplemental Figure 1). In quantitative comparisons, the HepG2-2B1 presented the highest mCherry expression on either percent (%) of fluorescent cells (Figure 1(B), upper panel) or mean fluorescence intensity (MFI, Figure 1(B), lower panel). Consistently, the HepG2-2B1 also showed the best PreS1-peptide binding performance among all test cell lines (Figure 1(C)). In addition, western blots also demonstrated the dox-treated 2B1 cells exhibited the highest expression level of NTCP (for both glycosylated and unglycosylated protein forms, Figure 1(D)).

In the evaluation of HBV susceptibility (Figure 1(E and F)), all cell lines successfully supported ccHBV infection at an MOI of 100 GE/cell, and no significant difference was observed in postinfection secretions of either HBeAg (Figure 1(E), lower panel) or HBsAg (Figure 1(F), lower panel). Comparison of the secretion levels of the two markers from HepG2-TetOn-NTCP cell lines at day 5 post infection (dpi 5) revealed that the average increase in the HBeAg titre (89-fold higher than the LLOQ) was significantly higher (*p* < 0.01) than that in the HBsAg titre (14-fold higher than the LLOQ). Similar results were noted when comparing these titres at dpi 9 (HBeAg, 688-fold higher than the LLOQ; HBsAg, 106-fold higher than the LLOQ; *p* < 0.01). When the cells were challenged with ccHBV at an extremely low MOI of 1 GE/cell (Figure 1(E and F), upper panels), they could also produce detectable HBeAg (4.3-fold higher than the LLOQ at dpi 9) but only showed very little HBsAg (1.5-fold higher than the LLOQ at dpi 9). Although both HBeAg and HBsAg are routinely used as HBV infection markers, these results suggested that HBeAg in culture supernatants was a more sensitive marker than HBsAg for evaluating HBV infection in HepG2-TetOn-NTCP cells. Similarly, other investigators found HepG2 cells overexpressing NTCP to secrete much more HBeAg than HBsAg following challenge with ccHBV of genotype D [12,16]. Therefore, the HBeAg level in the postinfection culture supernatant was considered the key parameter to quantitatively characterize HBV infection in our further study. Among the 5 cell lines we developed, as the HepG2-2B1 presented the best performance on PreS1-peptide binding capability, dox-inducible NTCP expression level and postinfection HBeAg secretion level (particularly at low-MOI virus challenge, Figure 1(E) upper panel), it was selected to establish the anti-HBV NAT assay in subsequent study.

### Establishment of an in vitro assay for analyzing HBV neutralization activity

**Figure 2.**
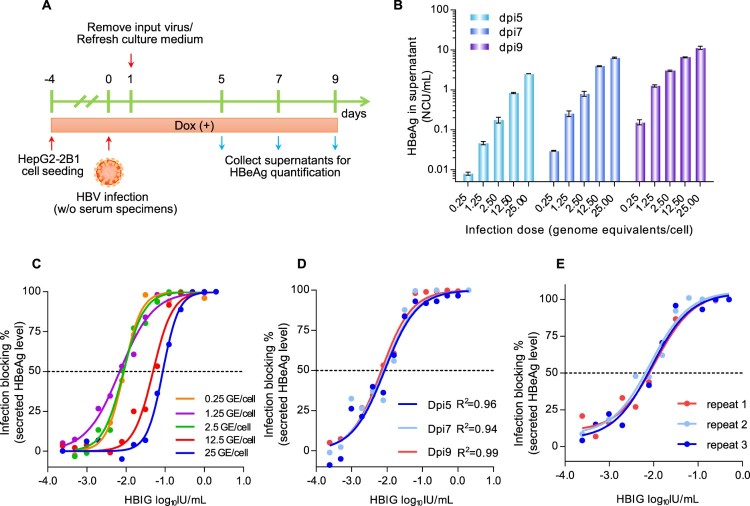
Establishment of a sensitive HBV neutralization assay in HepG2-2B1 cells. (A) A schematic diagram depicting the experimental procedure of the HBV NAT assay. (B) Comparisons of HBeAg secretion levels of HepG2-2B1 cells infected with ccHBV at different MOIs (0.25, 1.25, 2.5, 12.5, or 25 GE/cell) at dpi 5, 7 and 9. (C) Tests of the infection-neutralizing potential (inhibition ratio of HBeAg secretion by HBV-infected HepG2-2B1 cells at dpi 9) of HBIG at various HBV challenge doses (0.25, 1.25, 2.5, 12.5, or 25 GE/cell). (D) IC50 evaluation of the neutralizing potential of HBIG in HepG2-2B1 cells challenged with 1.25 GE/cell ccHBV at dpi 5, 7, and 9. The R-squared value was introduced to describe the goodness of fit. (E) Evaluation of the reproducibility of the NAT assay by using HBIG samples in three independent tests. For panels C, D and E, the curves were fitted by nonlinear regression (log [inhibitor] vs. normalized response).

Figure 2(A) illustrated the detailed procedure of the HepG2-2B1-based NAT assay. To optimize the assay performance, we evaluated the infection performance of HepG2-2B1 cells via ccHBV challenge at different MOIs (0.25, 1.25, 2.5, 12.5, or 25 GE/cell). The detection time point for HBeAg secretion was investigated at dpi 5, 7 and 9. As shown in Figure 2(B), quantifiable HBeAg (≥0.1 Ncu/mL) was found at dpi 5 in cells that received an HBV inoculum of ≥2.5 GE/cell. When the detection time point was extended to dpi 7 or dpi 9, quantifiable HBeAg levels were observed for HBV inocula of ≥1.25 GE/cell. To test the influence of the virus challenge dose on the sensitivity of NAT detection, we evaluated HBV infection at different MOIs in the presence of a 2-fold serial dilution of HBIG (a gradient of 14 concentrations from 2000 mIU/mL to 0.24 mIU/mL). The IC50 (half maximal inhibitory concentration for HBeAg secretion at dpi 9, Figure 2(C)) of HBIG to neutralize HBV was approximately 6.8–8.8 mIU/mL in groups that received a virus challenge of ≤2.5 GE/cell, significantly higher than that of cells challenged with 12.5 GE/cell (IC50 = 49.0 mIU/mL) or 25 GE/cell (IC50 = 87.1 mIU/mL). These results suggested that the analytical sensitivity of the NAT assay was negatively associated with the HBV challenge dose. When 1.25 GE/cell was selected as the standard MOI for the HBIG NAT assay, HBeAg measurements at dpi 5, 7 and 9 yielded quite similar dose–inhibition curves (Figure 2(D)). However, analyses at dpi 9 showed a better goodness-of-fit curve (*R*^2 ^= 0.99, Figure 2(D)). Moreover, the average HBeAg elevation at dpi 9 for a virus challenge of 1.25 GE/cell was 12.5-fold higher than the LLOQ (1.25 ± 0.08 Ncu/mL), which was higher than that at dpi 7 (2.6-fold higher than the LLOQ, 0.26 ± 0.04). Therefore, we ultimately selected an MOI = 1.25 GE/cell as the HBV challenge dose and dpi 9 as the detection time point for subsequent NAT measurements. Under these conditions, we evaluated the reproducibility of the NAT assay. As the data in Figure 2(E) show, three independent tests using HBIG samples exhibited very similar dose–inhibition curves and yielded very similar IC50 values (9.6 ± 1.3 mIU/mL). These results suggested good accuracy of the new assay.

### Association between the qAnti-HBs titre and the NAT titre in HBV-free individuals

A total of 164 serum samples from HBV-free individuals (negative for both HBsAg and HBV DNA) were used to investigate the associations between the anti-HBV NAT titre and other serological parameters. The characteristics of these individuals are presented in Table 1. The qAnti-HBs titres of these samples were measured with two independent commercial immunoassays. Correlation analysis revealed good consistency (*R*^2^ = 0.823, *p* < 0.001, kappa value = 0.84) between the Architect and Wantai qAnti-HBs assays (Supplemental Figure 2). Serial two-fold dilutions (2, 4, 8, 16, 32, 64, 128 and 256-fold dilutions in culture medium) of all samples were made to assess blocking effects against HBV infection (MOI = 1.25 GE/cell) in HepG2-2B1 cells. At dpi 9, the levels of secreted HBeAg were quantified to calculate the NAT titre, which was defined as the maximum fold dilution required to produce ≥50% inhibition (MDF_50_). For analyses, we divided these individuals into 4 groups according to their anti-HBs status and anti-HBc status as shown in Table 1. In either anti-HBc positive (group A and B) or negative (group C and D) subjects, individuals with detectable anti-HBs always had significantly higher NAT titres (group A vs. B, *p* < 0.001; group C vs. D, *p* = 0.001). Among anti-HBs positive subjects, although the average anti-HBs titre was similar (*p* = 0.50) in between anti-HBc positive (group A) and negative (group C) individuals, the average NAT titre was significantly higher in subjects who had detectable anti-HBc (*p* = 0.033). Similar results were also noted in anti-HBs negative donors (*p* = 0.030). We further compared the NAT titres of vaccinated anti-HBs positive individuals (*n* = 34) with nonvaccinated anti-HBc/anti-HBs double positive ones (*n* = 14) as shown in Supplemental Table 1. The results suggested that although the average anti-HBs binding titre was comparable (*p* = 0.39) between the two groups, the average NAT titre was higher in nonvaccinated anti-HBc/anti-HBs double positive individuals than that in vaccinated anti-HBs positive individuals (1.33 ± 0.60 v.s 0.94 ± 0.62, *p* = 0.0496). Overall, these results suggested that the NAT titres not only depend on anti-HBs but also associate with anti-HBc.

**Table 1.** Characteristics of the samples from HBV-free heathy subjects according to the serological anti-HBs and anti-HBc status.

Subgroup	All, *n* = 164	Anti-HBc(+)^a^, *n* = 119		Anti-HBc(−)^b^, *n* = 45		*P* value			
		Anti-HBs(+)^c^	Anti-HBs(−)^d^	Anti-HBs(+)	Anti-HBs(−)				
	Total	A	B	C	D	A vs. B	C vs. D	A vs. C	B vs. D
Number (%)	164 (100)	89 (54.3)	30 (18.3)	34 (20.7)	11 (6.7)				
Gender, M/F	112/52	57/32	21/9	25/9	9/2	0.66	0.70	0.39	0.69
Age, mean ± SD	26.9 ± 9.5	26.7 ± 9.1	25.8 ± 9.7	27.6 ± 9.9	29.1 ± 11.2	0.65	0.67	0.62	0.35
HB vaccination, n (%)	138 (84.1)	75 (84.3)	22 (73.3)	34 (100)	7 (63.6)	0.19	0.002	0.011	0.70
qAnti-HBs, log_10_^e^	1.72 ± 1.31	2.36 ± 0.76	−0.03 ± 0.84	2.26 ± 0.79	−0.34 ± 0.55	<0.001	<0.001	0.50	0.26
qAnti-HBc, log_10_^f^	−0.13 ± 1.11	0.51 ± 0.97	−0.34 ± 0.77	−1.26 ± 0.26	−1.31 ± 0.28	<0.001	0.62	<0.001	<0.001
NAT titre, log_10_^g^	0.97 ± 0.68	1.22 ± 0.67	0.52 ± 0.34	0.94 ± 0.62	0.25 ± 0.35	<0.001	0.001	0.033	0.030

Note: Anti-HBs, antibodies against hepatitis B surface proteins; Anti-HBc, antibodies against the hepatitis B core protein; qAnti-HBs, quantitative Anti-HBs; qAnti-HBc, quantitative Anti-HBc; NAT, HBV neutralization activity; ^a^Anti-HBc ≥ 0.1 IU/mL; ^b^Anti-HBc < 0.1^ ^IU/mL; ^c^Anti-HBs ≥ 10 mIU/mL; ^d^Anti-HBs < 10 mIU/mL; ^e^mIU/mL (mean ± SD); ^f^IU/mL (mean ± SD); ^g^the maximum fold dilution required to produce ≥50% inhibition (MDF_50_), data were mean ± SD.

We further analyzed the effect of the qAnti-HBs level on the distribution of NAT titre by stratifying all subjects into 5 groups according to their qAnti-HBs level (as determined by the Architect assay): <10 mIU/mL (*n* = 41), 10–50 mIU/mL (*n* = 31), 51–200 mIU/mL (*n* = 28), 201–1000 mIU/mL (*n* = 42), >1000 mIU/mL (*n* = 22). As shown in Table 2, there were 36 of 41 (87.8%) anti-HBs negative (<10 mIU/mL) samples had a NAT titre of MDF_50 _< 8. Among the remaining 5 samples with discordant results in the Architect qAnti-HBs assay and the NAT test, 1 sample had a NAT titre of MDF_50 _= 32; this sample was positive in the Wantai qAnti-HBs assay (17.8 mIU/mL) and the qAnti-HBc assay (42.3 IU/mL). The other 4 samples (Architect qAnti-HBs <10 mIU/mL, NAT MDF_50_ ≥ 8) were negative in the Wantai qAnti-HBs assay; however, 3 of these had detectable anti-HBc. Only one sample had a NAT titre of MDF_50_ ≥ 8 but was negative in both the Anti-HBs and Anti-HBc immunoassays. Therefore, the specificity of the NAT assay was 97.6% (95% confidence interval, 87.4–99.6%). On the other hand, among the 123 samples with a qAnti-HBs level greater than 10 mIU/mL (Architect assay), the NAT titres varied significantly and were widely distributed from 2 to 256 (MDF_50_ value); 87 of these (70.7%) showed a NAT titre of MDF_50_ ≥ 8 (Table 2). There were 12 (12/31, 38.7%) samples with an anti-HBs titre of 10–50 mIU/mL and 5 (5/28, 17.9%) samples with an anti-HBs titre of 51–200 mIU/mL did not show any detectable NAT activity (MDF_50 _< 2). In addition, 13 samples with an anti-HBs titre of 10–50 mIU/mL, 5 sample with an anti-HBs titre of 51–200 mIU/mL and 1 sample with anti-HBs titre of 201–1000 mIU/mL only presented very little NAT activity (MDF_50 _= 2).

**Table 2.** The distribution of NAT titres among the 164 serum samples according to the qAnti-HBs strata.

qAnti-HBs strata	Neutralizing Antibody Titer (NAT) strata								Total
	<2	4	8	16	32	64	128	256	
< 10 mIU/mL	14(7/7)	22(19/3)	4(3/1)	0	1(1/0)	0	0	0	41(30/11)
10–50 mIU/mL	12(9/3)	13(7/6)	4(3/1)	0	1(1/0)	1(1/0)	0	0	31(21/10)
51–200 mIU/mL	5(2/3)	5(3/2)	12(8/4)	2(2/0)	1(1/0)	3(3/0)	0	0	28(19/9)
201–1000 mIU/mL	0	1(1/0)	3(1/2)	10(7/3)	8(8/0)	15(13/2)	3(3/0)	2(2/0)	42(35/7)
>1000 mIU/mL	0	0	1(1/0)	2(2/0)	8(4/4)	5(2/3)	5(4/1)	1(1/0)	22(14/8)
Total	31(18/13)	41(30/11)	24(16/8)	14(11/3)	19(15/4)	24(19/5)	8(7/1)	3(3/0)	164(119/45)

Note: The data were number (Anti-HBc-positive no./Anti-HBc-negative no.). The NAT was defined as the maximum fold dilution required to inhibit HBeAg secretion by ≥50% (MDF_50_) in HepG2-2B1 cells infected with 1.25 GE/cell ccHBV at day 9 post-infection.

**Figure 3.**
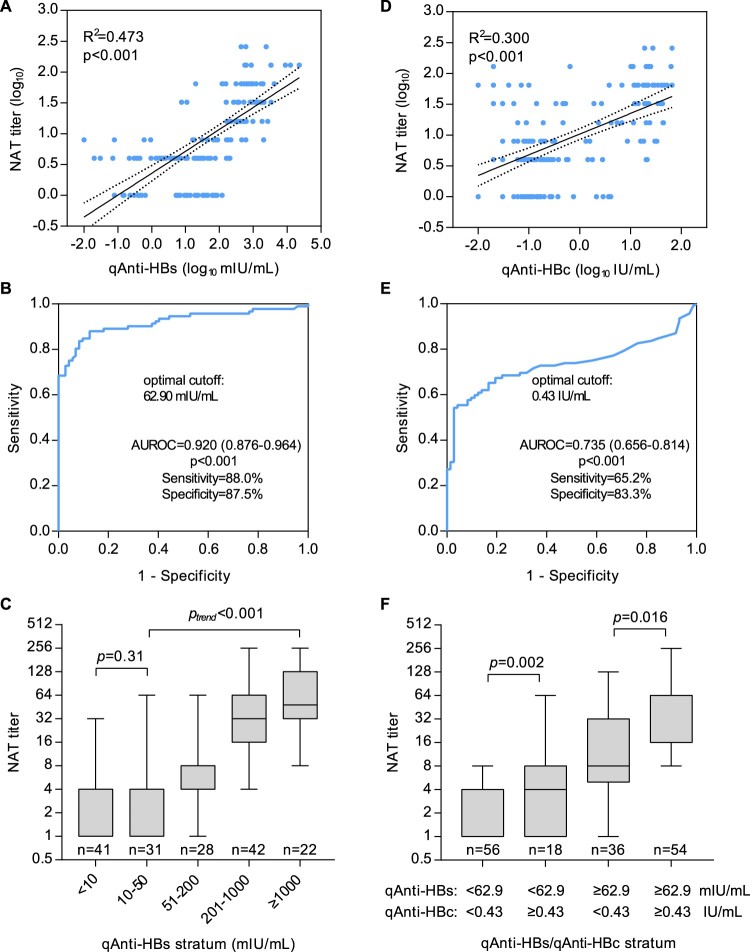
Association between the functional HBV neutralization titre and binding titres of anti-HBs and anti-HBc in sera from HBV-free heathy donors. (A) Correlation between the NAT and qAnti-HBs titres. (B) ROC analysis of the qAnti-HBs level to discriminate significant HBV-neutralizing activity (MDF_50_ ≥ 8 in NAT assay). (C) Average serum NAT titres in HBV-free subjects according to the qAnti-HBs stratum. (D) Correlation between the NAT and qAnti-HBc titres. (E) ROC analysis of the qAnti-HBc level to discriminate significant HBV-neutralizing activity (MDF_50_ ≥ 8 in the NAT assay). (F) Average serum NAT titres in HBV-free subjects according to the qAnti-HBs and qAnti-HBc strata. ROC, receiver operating characteristic curve; AUROC, area under the ROC curve.

In quantitative analyses, we found the NAT titres were strongly correlated with the qAnti-HBs levels (*R*^2 ^= 0.473, *p* < 0.001, Figure 3(A)) among all subjects as expected. When ROC analysis was performed to discriminate a NAT titre of MDF_50_ ≥ 8 using qAnti-HBs (Figure 3(B)), the AUROC was 0.920 (95% confidence interval, 0.876–0.964, *p* < 0.001), and the optimal cutoff was 62.9 mIU/mL, with a sensitivity of 88.0% and a specificity of 87.5%. As shown in Figure 3(C), the average NAT titre successively increased (p_trend_ < 0.001) with increasing qAnti-HBs levels among the subjects who had qAnti-HBs level of greater than 50 mIU/mL, but no significant difference was observed among the subjects whose qAnti-HBs level was <50 mIU/mL. Interestingly, we also observed a positive correlation between NAT titres and qAnti-HBc levels (*R*^2 ^= 0.300, *p* < 0.001, Figure 3(D)). The AUROC of qAnti-HBc for a NAT titre of MDF_50_ ≥ 8 was 0.735 (95% confidence interval 0.656–0.814, *p* < 0.001), and the optimal cutoff was 0.43 IU/mL, with a sensitivity of 65.2% and a specificity of 83.3% (Figure 3(E)). Further analyses revealed that individuals with a qAnti-HBc level of ≥0.43 IU/mL had significantly higher NAT titres than those who were anti-HBc negative or had a low level of qAnti-HBc (<0.43 IU/mL) in the groups with both high (*p* = 0.016) and low (*p* = 0.002) qAnti-HBs levels (Figure 3(F)).

## Discussion

The *in vitro* HBV neutralization assay is an important functional test to detect the presence and quantify the titre of systemic antibodies that prevent HBV infection. Usually, neutralizing antibodies that can interfere with the interaction between virus and its host cell receptor are merely part of a set of virus-specific antibodies in humans who received vaccine immunization or recovered from past virus infection [17]. Recombinant small HBsAg-based HBV vaccines have been widely used and can successfully stimulate neutralizing antibodies to prevent HBV infection in most individuals [1,4]. Due to the lack of a convenient and sensitive cell-based neutralization assay, serological tests for determining the titres of HBsAg-binding specific antibodies (anti-HBs) by immunoassays are commonly used for evaluating vaccine-induced protection strength as well as for indicating recovery and immunity from resolved HBV infection. However, previous studies have demonstrated that not all HBsAg-binding reactive antibodies have neutralizing activity [5]. Although a qAnti-HBs titre of ≥10 mIU/mL is considered to be the threshold for HBV seroprotection, the quantitative association between the qAnti-HBs titre and the NAT is still largely unknown. Moreover, the HBV NAT assay is a valuable tool for both virological studies and antiviral developments for treating HBV infection.

The major obstacle in developing a robust cell-based HBV NAT assay is the current low-efficiency *in vitro* HBV infection system [18]. Several cells and engineered cell lines, including primary human hepatocytes (PHHs) and primary Tupaia hepatocytes (PTHs), the HepaRG cell line, and NTCP-overexpressing hepatoma cell lines (HepG2-NTCP and Huh7-NTCP), have been demonstrated to be HBV-susceptible [16,19,20]. However, the infection efficiency and cell source of primary hepatocytes are limited, and HepaRG cells require a time-consuming differentiation culture process (approximately 4 weeks) [21]. In recent years, NTCP-overexpressing hepatoma cells have been an important advancement in the development of high-throughput infection assays, but it is important to note that a high-MOI (ranging from 100 to 10,000) virus challenge is still required to attain acceptable infection levels for nearly all of the previously reported NTCP-reconstituted hepatoma cell lines [16,18–20]. For the NAT assay, a high-MOI virus challenge greatly reduces the analytical sensitivity for the detection of neutralizing antibodies (Figure 2(C)). As qAnti-HBs levels vary significantly and are widely distributed among humans, a robust assay for the direct measurement of the NAT titre in serum specimens should support HBV infection in challenges with low-MOI viral inocula. In this study, we established a new HepG2 cell clone (HepG2-2B1) allowing dox-regulated expression of human NTCP. In these cells, the dox-inducible NTCP expression cassette was stably integrated into the HepG2 cell genome downstream of an IRES-mCherry reporter, therefore enabling FACS selection of cell clones with high expression levels of NTCP via the evaluation of mCherry fluorescence. The HepG2-2B1 cell clone showed more stable and higher levels of NTCP expression (Figure 1(D)) and PreS1-peptide binding capability (Figure 1(C)), which possibly contributed to its better infection performance than that of the other clones, particularly in cells infected with a low-MOI HBV inoculum (Figure 1(E and F)). By using this HepG2-2B1-based infection system, we investigated the optimal virus inoculum dose (1.25 GE/cell) and detection time point (dpi 9) for neutralization analysis and established a robust NAT assay (Figure 2). The new assay showed excellent analytical sensitivity (9.6 ± 1.3 mIU/mL of HBIG equivalent) and good accuracy (Figure 2(E)), therefore providing a possible tool enabling the direct measurement of human serum samples. On the other hand, unlike other cells with exogenous NTCP expression driven by constitutive promoter, HepG2-2B1 allows tightly regulable NTCP expression by dox. As the strength of TRE3G promoter is highly dox dose-dependent, the new cell line may enable to control the NTCP at various levels, giving more flexibility over other constitutively NTCP-expressing systems.

We quantitatively analyzed the relationship between HBV serological markers and serum NAT titres in a cohort of HBV-free healthy donors. As expected, our data revealed a strong positive correlation between the qAnti-HBs and NAT titres (Figure 3(A), *R*^2 ^= 0.473). However, 29.3% (36/123) of persons with qAnti-HBs ≥ 10 mIU/mL (ranging from 10.7 to 202.3 mIU/mL) did not present significant NAT activity (MDF_50_ ≥ 8). Among these subjects, 17 showed no detectable NAT (MDF_50 _< 2) and the remaining 19 had very little NAT (MDF_50 _= 4) (Table 2). These results suggested that some human anti-HBs antibodies might not be able to block HBV infection although they can bind to HBsAg proteins. As most of these samples (25/36, 69.4%) had a relatively low anti-HBs titre (10–50 mIU/mL), the low-affinity of antibodies existing in such samples may be one possible reason for the NAT absence. There was one sample with a high anti-HBs titre of 202.3 mIU/mL showing very little NAT activity (MDF50 = 4). As this sample is anti-HBc seropositive, its low NAT activity may be attributed to past infection with a virus which had mutated surface proteins and was different than ccHBV used our assay. Notably, a recent study demonstrated that a qAnti-HBs titre of 56.48  mIU/mL is a protective threshold (hazard ratio = 8.48) for HBV reactivation risk in patients with lymphoma and resolved HBV infection receiving rituximab-containing chemotherapy [22]. Our analyses suggested that an optimal cutoff value of the qAnti-HBs level to discriminate significant NAT (MDF_50_ ≥ 8) was 62.9 mIU/mL, which was quite similar to the newly determined protective threshold for HBV reactivation, and both of these values were much higher than the commonly used protective threshold (10 mIU/mL). These results suggested that sufficient HBV protective immunity may require a higher anti-HBs binding titre, possibly greater than 50–60 mIU/mL. Interestingly, our analyses revealed that the anti-HBs and anti-HBc double positive donors had similar anti-HBs titres but showed significantly higher NAT titres than those who were anti-HBs positive but anti-HBc negative (Table 1). Moreover, the qAnti-HBc level was also independently associated with serum NAT (*R*^2 ^= 0.300, *p* < 0.001, Figure 3(D)). Because anti-HBc is a serological marker of past, resolved HBV infection, it is possible that the appearance of anti-HBc is a proxy for anti-HBV immunity derived from non-HBsAg neutralizing antibodies (such as PreS1/PreS2-specific antibodies). This hypothesis warrants further study to determine whether there is an association between serum NAT activity and PreS1/PreS2-specific antibodies among anti-HBc positive individuals. On the other hand, the possibility that a high titre of anti-HBc antibody has direct HBV-neutralization activity or a potential interference effect on the current NAT assay cannot be entirely excluded.

To our knowledge, this study is the first to develop a cell-based assay enabling measurements of HBV NAT in human serum samples. By using this new assay, we provided direct evidence for the relationship between the anti-HBs binding titre and functional activity in HBV-free healthy persons. A limitation of our study is the low case number, and our findings need to be confirmed in a larger cohort. In addition, we just evaluated the assay performance in HBV-free heathy individuals but not in HBV patients. Further improvements may be required for its real clinical application. Nevertheless, our study provides a versatile platform for testing the efficacy of new drug candidates, as well as a valuable tool for analyzing functional antibodies of clinical samples, in particular for patients with coexisting HBsAg and anti-HBs.

## Supplementary Material

Supplemental Material
