# Supplementary material for: Robust in vitro assay for analyzing the neutralization activity of serum specimens against hepatitis B virus
Source: Emerg Microbes Infect. 2019 May 25;8(1):724–33. doi: 10.1080/22221751.2019.1619485 (PMC6542156; doi:10.1080/22221751.2019.1619485)
Supplement: Supplemental Material [file TEMI_A_1619485_SM5629.docx]

Supplemental Figure 1. Original data of flow cytometric analyses of the dox-induced mCherry expression and dose-dependent PreS1-peptide (FITC) binding performance of the different HepG2-TetOn-NTCP cell lines.

Supplemental Figure 2. Correlation between the anti-HBs titers determined by two commercial immunoassays.


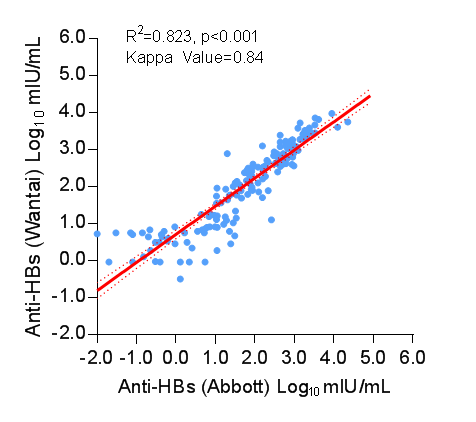


Supplemental Table 1. Comparison of vaccinated anti-HBs positive individuals with nonvaccinated anti-HBc/anti-HBs double positive individuals

|  | Anti-HBs only derived from past HBV infection | Anti-HBs only derived from Vaccination | P value |
| --- | --- | --- | --- |
| Vaccination | No | Yes |  |
| Anti-HBc status | Positive | Negative |  |
| Anti-HBs status | >10 mIU/mL | >10 mIU/mL |  |
| Number | 14 | 34 |  |
| Gender, M/F | 9/5 | 25/9 | 0.73 |
| Age, mean±SD | 41.8±7.7 | 27.6±9.9 | <0.001 |
| qAnti-HBs, log_10_ | 2.48±0.84 | 2.26±0.79 | 0.39 |
| qAnti-HBc, log_10_ | 2.54±0.79 | -1.26±0.26 | <0.001 |
| NAT titer, log_10_ | 1.33±0.60 | 0.94±0.62 | 0.0496 |
